# Comparison of glycemic control and β-cell function in new onset T2DM patients with PCOS of metformin and saxagliptin monotherapy or combination treatment

**DOI:** 10.1186/s12902-018-0243-5

**Published:** 2018-02-27

**Authors:** Tao Tao, Peihong Wu, Yuying Wang, Wei Liu

**Affiliations:** 1 Department of Endocrinology and Metabolism, Renji Hospital, School of Medicine, Shanghai Jiaotong University, 160 Pujian Road, Shanghai, 200127 China; 2 Shanghai Key laboratory for Assisted Reproduction and Reproductive Genetics, Center for Reproductive Medicine, Renji Hospital, School of Medicine, Shanghai Jiaotong University, 160 Pujian Road, Shanghai, 200127 China

**Keywords:** Polycystic ovary syndrome, Type 2 diabetes mellitus, Saxagliptin, Metformin

## Abstract

**Background:**

Impaired insulin activity in women with polycystic ovary syndrome might differ from that seen in type 2 diabetes mellitus without polycystic ovary syndrome. This study was designed to compare the effects of treatment with metformin, saxagliptin, and their combination in newly diagnosed women with type 2 diabetes mellitus and polycystic ovary syndrome in China.

**Methods:**

A total of 75 newly diagnosed patients from Shanghai, China with type 2 diabetes mellitus and polycystic ovary syndrome were included in this randomized, parallel, open-label study. All patients received treatment for 24 weeks with metformin, saxagliptin, or their combination. Patients were allocated to one of three treatment groups by a computer-generated code that facilitated equal patient distribution of 25 patients per group. The primary outcome was a change in glycemic control and β-cell function.

**Results:**

A total of 63 patients completed the study (*n* = 21, for each group). The reduction in hemoglobin A1c was significant in the combination group, compared to the monotherapy groups (saxagliptin vs. combination treatment vs. metformin: − 1.1 vs. -1.3 vs. -1.1%, *P* = 0.016), whereas it was comparable between the metformin and saxagliptin groups (*P* > 0.05). Saxagliptin, metformin, and the combination treatment significantly reduced the homeostasis model assessment- insulin resistance index and increased the deposition index (*P* < 0.01 for all). However, no significant change was observed in the homeostasis model assessment- β-cell function among the metformin and combination groups, and no significant changes were observed in the insulinogenic index among all three groups (*P* > 0.05 for all). In addition, saxagliptin and metformin treatments significantly reduced body mass index and high-sensitivity C-reactive protein levels (*P* < 0.01 for both).

**Conclusions:**

Saxagliptin and metformin were comparably effective in regulating weight loss, glycemic control, and β-cell function, improving lipid profiles, and reducing inflammation in newly diagnosed type 2 diabetes mellitus patients with polycystic ovary syndrome.

**Trial registration:**

ChiCTR-IPR-17011120 (retrospectively registered on 2017–04-12).

**Electronic supplementary material:**

The online version of this article (10.1186/s12902-018-0243-5) contains supplementary material, which is available to authorized users.

## Background

Polycystic ovary syndrome (PCOS) affects 6–10% of reproductive-age women. Insulin resistance (IR) and hyperinsulinemia play a significant role in the predisposition to diabetes in PCOS [1]. About 30–40% of obese reproductive-age women with PCOS have impaired glucose tolerance (IGT) [1, 2], and approximately 10% have type 2 diabetes mellitus (T2DM) based on a 2-h glucose level > 200 mg/dL [3]. Notably, only a small fraction of women with PCOS and either IGT or T2DM display fasting hyperglycemia that is consistent with diabetes, based on the American Diabetes Association criteria.

The findings of Dunaif and coworkers [4] suggested that the impaired insulin activity in women with PCOS might differ from that seen in T2DM without PCOS, or in obese women, who did not exhibit the classical features of PCOS. Our previous study [5] reported early impairment of β-cell function in women with PCOS. Moreover, a more serious primary defect in insulin action has been detected in lean women with PCOS, compared to obese women with PCOS in China [5]. Therefore, reduced insulin secretion, particularly during the first phase of secretion, is the main characteristic of newly diagnosed women with PCOS and T2DM. However, the exact mechanism associated with this attenuated β-cell function in women with PCOS remains unclear. Recent studies have shown that an incretin defect might be related to β-cell dysfunction [6]. An important consideration is raised about the manner in which interventions might effectively treat hyperglycemia in women with T2DM and PCOS.

Metformin inhibits hepatic glucose production and increases peripheral glucose uptake and utilization [7]. Metformin can both improve insulin sensitivity in target tissues and directly influence ovarian steroidogenesis, and these effects do not appear to be primarily responsible for the attenuation of ovarian androgen production in women with PCOS [8, 9]. Although metformin benefits patients with diabetes by improving insulin sensitivity, whether it increases insulin secretion, particularly during the first phase of secretion, remains unclear.

Saxagliptin is thought to exert its effects by delaying the inactivation of incretin, through the inhibition of the dipeptidyl peptidase-4 (DPP-4) inhibitor, thereby enhancing and prolonging the action of incretin. This results in improved glucose-mediated insulin release and reduced postprandial glucagon secretion [6]. However, only a few studies have compared the effects of metformin and saxagliptin on glycemic control in patients with new-onset T2DM and PCOS. Therefore, an important question was raised of whether other medicines that modulate glycemic control might show more optimal effects than metformin in preventing the development of diabetes in Chinese women with PCOS.

This study therefore aimed to compare the effects of metformin and saxagliptin monotherapy, and metformin and saxagliptin combination therapy on blood glucose, hemoglobin A1c (HbA1c), anthropometric measurements, lipid profiles, and inflammation in newly diagnosed women with T2DM and PCOS.

## Methods

### Study design and patients

This study was an open-label prospective, randomized clinical trial conducted over 24 weeks, with three treatment groups. The primary outcome was a change in glycemic control and β-cell function. A total of 75 newly diagnosed patients with T2DM and PCOS were included in the study. They were recruited from the Outpatient Department of Endocrinology and Metabolism at Shanghai Renji Hospital. The PCOS diagnosis was based on the Rotterdam Criteria (2003), and T2DM was diagnosed based on the World Health Organization criteria (1998). Patients with coronary atherosclerotic heart disease, abnormal liver and renal function, diabetic ketoacidosis, chronic inflammatory disease, and severe gastrointestinal disease were excluded. All participants had a control diet for 2 weeks before treatment. Patients were allocated to one of three treatment groups by a computer-generated code that facilitated equal patient distribution of 25 patients per group. The study protocol was approved by the Human Research Ethics Committee of the Shanghai Renji Hospital, and written informed consents were obtained from all study participants (Clinical trial registration number: ChiCTR-IPR-17011120). All study evaluations and procedures were conducted in accordance with the guidelines of the Helsinki Declaration on human experimentation.

All participants were given advice on diet and exercise and were asked to follow a behavior modification program. Twenty-one patients received metformin (2000 mg/day), 21 patients received saxagliptin (5 mg/day), and 21 patients received combination therapy of metformin (2000 mg/day) and saxagliptin (5 mg/day). The administration of metformin or saxagliptin was fixed throughout the 24-week treatment period.

All patients were asked to presented to the Department of Endocrinology and Metabolism at Shanghai Renji Hospital at baseline and after the 24-week treatment period. Before the study day, patients were asked to have their dinner before 6 p.m. After the meal, patients were asked to fast for 14 h from solids and 12 h for liquids until the morning of the study day. On the study day, blood samples of the participants were collected at 8 a.m. for measurements of blood glucose, HbA1c, insulin, lipids, and high-sensitivity C-reactive protein (hsCRP). Height and weight were measured at baseline and at the end of treatment. Each patient completed a checklist and received weekly telephone contact to assess compliance after taking the medication.

### Measurements

#### Anthropometric measurements

The height and weight of each subject were measured in light clothing to the nearest 1 cm and 0.1 kg, respectively. The waist circumference (WC) and hip circumference (HC) were measured by a particular investigator. The WC was measured at the narrowest circumference between the lower border of the rib cage and the iliac crest. The HC was measured at the level of the symphysis pubis and the greatest gluteal protuberance. Body mass index (BMI) = body weight (kg) / height (m) squared. The waist hip ratio (WHR) = WC (cm) / HC (cm). Weight, WC, and HC were measured at baseline and after the 24-week treatment.

#### Oral glucose tolerance test (OGTT) and relevant calculations

All study participants underwent a standard OGTT with 75 g glucose. The measurements for participants with PCOS were taken at baseline and at the end of treatment. After at least 8 h overnight fasting, blood samples were drawn to determine glucose and insulin levels before the glucose load, and they were again drawn at 30, 60, 120, and 180 min to determine the respective levels at those time points (marked as Gx, and Ix, where G was glucose and I was insulin).

### Laboratory analysis

Blood glucose levels were measured by hexokinase method. Insulin concentrations were measured by a radioimmunoassay kit (Beijing Atom HighTech Co. Ltd., Beijing, China). The intra-assay coefficients of variation (CV) of insulin were 5.5%. The HbA1c levels were measured using high-pressure liquid chromatography. The lipid profile levels were measured on a clinical chemistry analyzer (Roche Original Reagents, Stockholm, Sweden). Analysis of the hsCRP was performed using immunonephelometric methods and a BN-II analyzer (Dade Behring, Deerfield, Germany). The inter- and intra-assay CV were 4.9 and 6.8%, respectively. Competitive electrochemiluminescence immunoassays on the Elecsys autoanalyzer 2010 (Roche Diagnostics, IN, USA) were used to quantify serum total testosterone (T), luteinizing hormone (LH), and follicle-stimulating hormone (FSH). The intra-assay CV of insulin and steroid hormone assays were < 10%. Sex hormone binding globulin (SHBG) levels were measured by chemiluminescent immunoassay (Elecsys autoanalyzer 2010, Roche Diagnostics), validated for plasma SHBG. The CV for SHBG using this methodology was 6%. Free androgen indexes (FAI) were calculated based on T and SHBG levels, i.e.: FAI = T / SHBG × 100.

### Calculations

1. Insulin resistance was calculated by the homeostasis model assessment- insulin resistance index (HOMA-IR) [10] as follows:

$$ \mathrm{fasting}\ \mathrm{insulin}\ \left(\upmu \mathrm{IU}/\mathrm{mL}\right)\times \mathrm{fasting}\ \mathrm{plasma}\ \mathrm{glucose}\ \left(\mathrm{mmol}/\mathrm{L}\right)/22.5. $$

2) Whole-body insulin sensitivity was calculated by the Matsuda index [11] as follows:

$$ \mathrm{Matsuda}\ \mathrm{index}=10\ 000/\surd \left[\left(\mathrm{fasting}\ \mathrm{glucose}\times \mathrm{fasting}\ \mathrm{insulin}\right)\times \left(\mathrm{mean}\ \mathrm{glucose}\times \mathrm{mean}\ \mathrm{insulin}\ \mathrm{during}\ \mathrm{OGTT}\right)\right] $$

3) Islet β-cell function was evaluated by the homeostasis model assessment- β-cell function (HOMA-IS) [10] as follows:

$$ \Big\{20\times \mathrm{fasting}\ \mathrm{insulin}\ \left(\upmu \mathrm{IU}/\mathrm{mL}\right)/\left(\mathrm{fasting}\ \mathrm{plasma}\ \mathrm{glucose}\ \left(\mathrm{mmol}/\mathrm{L}\right)-3.5\right). $$

4) The insulinogenic index (ΔI_30_/ΔG_30_) (mIU/mmol) that is indicative of early-phase insulin secretion was calculated [11] as follows:

$$ \left(\mathrm{I}30-\mathrm{I}0\right)/\left(\mathrm{G}30-\mathrm{G}0\right). $$

5) The responses in glucose and insulin to the glucose load were also assessed by calculating the area under the curve during the OGTT for glucose (AUC glucose) and insulin (AUC insulin), respectively, using the trapezoidal rule [12].
6) The deposition index (DI) was calculated to estimate the β-cell response, relative to the prevailing insulin sensitivity [13], i.e.:

$$ \mathrm{DI}=\Delta \mathrm{I}30/\Delta \mathrm{G}30\ \left(\mathrm{mIU}/\mathrm{mmol}\right)/\mathrm{HOMA}-\mathrm{I}\mathrm{R}=\left(\mathrm{I}30-\mathrm{I}0\right)/\left(\mathrm{G}30-\mathrm{G}0\right)/\mathrm{HOMA}-\mathrm{I}\mathrm{R}. $$

### Sample size and statistical analysis

To our knowledge, there were no previous studies using the DPP-4 inhibitor in the treatment of patients with PCOS when this clinical trial was first proposed. Thus, a non-inferiority trial was designed, with an average standard deviation (SD) of 0.22, that required 21 completers per treatment group, to yield a power of 90% to detect a statistically significant difference (α = 0.05). The study was designed to recruit 25 patients in each group, based on an assumed dropout rate of 20%. Thus, 75 patients (25 per group) were required for random assignment.

The analysis was conducted in the per-protocol population (saxagliptin, *n* = 21; metformin, *n* = 21; and combination, *n* = 21). All statistical analyses were performed using SPSS version 21 (Statistical Package for the Social Sciences, USA). The normality of all variables was checked using the Shapiro–Wilk test. The results were presented as mean ± SD for variables of normal distribution, and mean (95% CI) for variables of skewed distribution. Statistical comparisons were made using one-way ANOVA for differences among the three groups, the paired *t*-test for changes observed in variables of normal distribution before and after treatment, and the Wilcoxon signed-rank test for variables of skewed distribution for differences between baseline and after the 24-week treatment. The Kruskal–Wallis test was used for variables of skewed distribution and one-way ANOVA was used for variables of normal distribution to evaluate differences among the three groups, and the Mann–Whitney U test was used to evaluate differences between the monotherapy groups. Statistical significance was set at *P* < 0.05.

## Results

### Clinical and biochemical patterns of target patients

Although 75 patients were randomly divided into three groups, 63 patients completed the 24-week treatment (saxagliptin, *n* = 21; metformin, *n* = 21; combination, *n* = 21), owing to migration, poor compliance, and adverse events. An additional file shows the numbers and characteristics of the various participants in more detail (see Additional file 1). The clinical characteristics and biochemical variables for the three groups according to the different therapies are summarized in Table 1. As expected, there were no significant differences in age, body weight, BMI, WC, WHR, or body fat (FAT)% among the three groups (*P* > 0.05 for all). Furthermore, the fasting blood glucose (FBG), 2-h glucose (2hBG), fasting insulin (FINS), 2-h insulin (2hINS), HbA1c, AUC glucose, and AUC insulin values showed no significant differences among the various groups (*P* > 0.05 for all). With respect to the lipid profile and inflammation, no significant differences were observed in triglyceride (TG), total cholesterol (TC), high-density lipoprotein cholesterol (HDL-C), low-density lipoprotein cholesterol (LDL-C), and hsCRP levels among the three groups (*P* > 0.05 for all). Moreover, sex hormone parameters, including LH, FSH, T, SHBG, and FAI showed no significant differences among the three groups (*P* > 0.05 for all).

**Table 1:** Baseline characteristics in PCOS patients with new-onset type 2 diabetes

Parameters	Saxagliptin	Saxagliptin + Metformin	Metformin	*P-*value
N	21	21	21	N/A
Age, years	30 ± 5	29 ± 5	28 ± 3	0.131
Weight, kg	70.4 (63.7–77.1)	69.3 (64.6–74.1)	67.9 (63.6–72.2)	0.886
BMI, kg/m^2^	27.2 (24.94–29.46)	26.38 (24.66–28.1)	26.4 (24.63–28.18)	0.904
WC, cm	86.8 (81.2–92.4)	84.7 (80.0–89.4)	82.8 (79.0–86.6)	0.395
WHR	0.88 ± 0.08	0.86 ± 0.06	0.85 ± 0.06	0.256
FAT%	36.13 (32.74–39.53)	35.12 (32.19–38.05)	33.6 (31.21–35.98)	0.397
FBG, mmol/L	5.63 (5.31–5.96)	5.84 (5.62–6.06)	5.62 (5.39–5.84)	0.166
2hBG, mmol/L	14.73 (13.49–15.97)	15.59 (14.26–16.92)	14.64 (13.66–15.62)	0.482
FINS, μIU/mL	15.78 ± 6.7	16.18 ± 5.3	14.34 ± 4.85	0.546
2hINS, μIU/mL	100.24 (80.63–119.85)	111.82 (92.73–130.91)	100.26 (84.59–115.94)	0.298
HbA1c, %	7.4 ± 0.3	7.4 ± 0.3	7.3 ± 0.2	0.668
AUC glucose	16.63 (15.65–17.62)	17.18 (16.16–18.21)	16.5 (15.81–17.19)	0.586
AUC insulin	129.93 (105.47–154.39)	148.2 (128–168.41)	122.66 (104.04–141.27)	0.152
TG, mmol/L	1.44 (1.16–1.72)	1.34 (1.19–1.5)	1.32 (1.05–1.59)	0.634
TC, mmol/L	4.51 (4.2–4.81)	4.8 (4.44–5.15)	4.94 (4.57–5.31)	0.175
HDL-C, mmol/L	1.26 (1.16–1.37)	1.24 (1.14–1.35)	1.35 (1.26–1.43)	0.167
LDL-C, mmol/L	3.06 ± 0.7	3.4 ± 0.73	3.32 ± 0.69	0.273
hsCRP, mg/L	3.97 (3.1–4.84)	3.94 (3.09–4.8)	4.02 (3.23–4.8)	0.937
LH, IU/L	14.2 (11.28–17.12)	11.57 (9.76–13.37)	12.53 (10.14–14.92)	0.434
FSH, IU/L	6.51 ± 2.04	6.76 ± 1.44	7.33 ± 2.36	0.393
T, nmol/L	2.64 ± 0.69	2.65 ± 0.67	2.64 ± 0.69	0.999
SHBG, nmol/L	24.72 (17.14–32.29)	30.1 (20.48–39.72)	22.64 (15.73–29.54)	0.382
FTI	15.82 (11.06–20.58)	11.72 (8.36–15.08)	16.91 (11.22–22.6)	0.274

Data are presented as mean (95% CI); age, WHR, HbA1c, and LDL-C are presented as mean ± SD

*P*-values are based on one-way ANOVA for variables of normal distribution and the Kruskal–Wallis test for variables of skewed distribution for differences among three groups

*BMI* body mass index, *WC* waist circumference, *WHR* waist–hip ratio, *FAT%* body fat percentage, *FBG* fasting blood glucose, *2hBG* 2-h glucose, *FINS* fasting insulin, *2hINS* 2-h insulin, *HbA1c* hemoglobin A1c, *AUC glucose* glucose area under the curve during oral glucose tolerance test (OGTT), *AUC insulin* insulin area under the curve during OGTT, *TG* triglyceride, *TC* total cholesterol, *HDL-C* high-density lipoprotein cholesterol, *LDL-C* low-density lipoprotein cholesterol, *hsCRP* high-sensitivity C-reactive protein, *LH* luteinizing hormone, *FSH* follicle-stimulating hormone, *T* total testosterone, *SHBG* sex hormone binding globulin, *FTI* Free testosterone index

### Changes in parameters of glucose metabolism after saxagliptin, metformin, or combination treatment in patients with new-onset T2DM

Table 2 presents glucose metabolism parameters in the saxagliptin, metformin, and combination therapy groups. Significant reductions in HbA1c were observed in all three groups after 24 weeks of treatment (*P* < 0.001 for all). The decline in HbA1c was more significant in the combination group, compared to the monotherapy groups, whereas differences between the monotherapy groups were not significant (saxagliptin vs. combination vs. metformin: − 1.1% vs. -1.3% vs. -1.1%, respectively, *P* = 0.016; saxagliptin vs. metformin: *P* = 0.890).

**Table 2:** Parameters of glucose metabolism before and after treatment in PCOS patients

Parameters	Saxagliptin			Saxagliptin + Metformin			Metformin		
	Baseline	Treatment	Δ	Baseline	Treatment	Δ	Baseline	Treatment	Δ
FBG, mmol/L	5.63 (5.31 to 5.96)	5.39 (5.15 to 5.62)*	−0.25 (− 0.49 to 0)	5.84 (5.62 to 6.06)	5.14 (5 to 5.27)**	− 0.7 (− 0.94 to − 0.46)	5.62 (5.39 to 5.84)	5.01 (4.88 to 5.14)**	−0.61 (− 0.86 to 0.35)
2hBG, mmol/L	14.73 (13.49 to 15.97)	7.23 (6.61 to 7.85)**	−7.5 (− 8.62 to − 6.38)	15.59 (14.26 to 16.92)	7.4 (6.93 to 7.86)**	−8.2 (− 9.5 to − 6.9)	14.64 (13.66 to 15.62)	7.78 (7.36 to 8.2)**	−6.86 (− 8.02 to − 5.7)
FINS, μIU/mL	15.78 ± 6.7	11.55 ± 4.57**	− 4.23 ± 3.86	16.18 ± 5.3	10.67 ± 2.99**	−5.51 ± 2.92	14.34 ± 4.85	10.26 ± 1.74**	− 4.08 ± 4.45
2hINS, μIU/mL	100.24 (80.63–119.85)	63.24 (49.57–76.91)**	−37 (− 49.94 to − 24.06)	111.82 (92.73 to 130.91)	69.49 (58.84 to 80.14)**	− 42.33 (− 57.39 to − 27.27)	100.26 (84.59 to 115.94)	61.15 (49.87 to 72.43)**	−39.11 (− 53.1 to − 25.13)
HbA1c, %	7.4 ± 0.3	6.3 ± 0.2**	− 1.1 ± 0.4*	7.4 ± 0.3	6.1 ± 0.2**	− 1.3 ± 0.3*	7.3 ± 0.2	6.3 ± 0.3**	−1.1 ± 0.4*
AUC glucose	16.63 (15.65 to 17.62)	11.7 (10.82 to 12.57)**	−4.94 (− 5.62 to − 4.26)	17.18 (16.16 to 18.21)	11.53 (10.92 to 12.14)**	−5.66 (− 6.44 to − 4.87)	16.5 (15.81 to 17.19)	11.19 (10.72 to 11.66)**	−5.31 (− 6.07 to − 4.56)
AUC insulin	129.93 (105.47 to 154.39)	98.75 (83.41 to 114.1)**	−31.18 (− 44.49 to − 17.87)	148.2 (128 to 168.41)	120.77 (109.13 to 132.4)**	−27.44 (− 41.07 to − 13.81)	122.66 (104.04 to 141.27)	88.67 (76.72 to 100.62)**	−33.99 (− 48.58 to − 19.4)
HOMA-IR	4.03 (3.11 to 4.95)	2.82 (2.21 to 3.42)**	−1.21 (− 1.71 to − 0.71)	4.22 (3.55 to 4.89)	2.45 (2.1 to 2.8)**	−1.77 (− 2.17 to − 1.38)	3.56 (3.01 to 4.11)	2.29 (2.1 to 2.47)**	−1.28 (− 1.82 to − 0.74)
HOMA-IS	157.2 (123.12 to 191.28)	125.36 (101.75 to 148.96)*	−31.85 (− 61.16 to − 2.54)	141.38 (119.5 to 163.25)	133.3 (115.27 to 151.33)	−8.07 (−30.3 to 14.15)	143.91 (116.96 to 170.86)	141.32 (121.93 to 160.71)	−2.59 (− 28.77 to 23.58)
Insulinogenic Index	21.04 (15.19 to 26.89)	20.32 (15.72 to 24.91)	−0.72 (− 4.99 to 3.54)	23.65 (18.11 to 29.19)	22.57 (18.23 to 26.91)	−1.08 (− 5.65 to 3.49)	19.35 (14.84 to 23.87)	18.76 (15.23 to 22.29)	−0.6 (− 6.15 to 4.96)
Matsuda Index	50 (39.81 to 60.18)	78.6 (63.89 to 93.32)**	28.61 (21.58 to 35.63)	42.41 (35.12 to 49.7)	71.37 (62.45 to 80.29)**	28.96 (25.24 to 32.69)	50.12 (44.35 to 55.89)	84.82 (76.28 to 93.37)**	35.26 (27.86 to 42.66)
DI	6.1 (4.42 to 7.77)	8.7 (6.2 to 11.19)**	2.6 (0.59 to 4.62)	5.99 (4.5 to 7.49)	9.57 (8.11 to 11.03)**	3.57 (1.81 to 5.34)	5.63 (4.23 to 7.02)	8.71 (6.29 to 11.13)**	3.09 (0.53 to 5.64)

Data are presented as mean ± SD or mean (95% CI). *P*-values are based on the paired *t*-test for variables of normal distribution and the Wilcoxon signed-rank test for variables of skewed distribution, for differences between baseline and after the 24-week treatment; one-way ANOVA for variables of normal distribution and the Kruskal–Wallis test for variables of skewed distribution for differences among three groups. Δ denotes the changes after treatment compared with baseline. * *P* < 0.05 and ** *P* < 0.01 for changes before and after treatment in treatment columns, and for changes among three groups in Δ columns

*FBG* fasting blood glucose, *2hBG* 2-h blood glucose, *FINS* fasting insulin, *2hINS* 2-h insulin, *HbA1c* hemoglobin A1c, *AUC glucose* glucose area under the curve during oral glucose tolerance test (OGTT), *AUC insulin* insulin area under the curve during OGTT, *HOMA-IR* homeostasis model assessment of insulin resistance, *HOMA-IS* homeostasis model assessment of insulin secretion, *DI* deposition index

Parameters reflective of β-cell function are also presented in Table 2. The DI, insulinogenic index, and HOMA-IS, the parameters of β-cell function, were estimated both before and after the 24-week treatment. The insulinogenic index in the three groups and the HOMA-IS in the combination group and metformin group showed no significant change after the 24-week treatment (*P* > 0.05 for all), whereas the HOMA-IS in the saxagliptin group showed a significant decline (*P* = 0.046). Furthermore, an improvement was observed in the DI of all three groups after 24 weeks of treatment (saxagliptin group: *P* = 0.004; combination group: *P* = 0.001; metformin group: *P* = 0.003).

Patients in all three groups exhibited improved insulin sensitivity, which was indicated by the HOMA-IR and Matsuda index (*P* < 0.001 for all). Changes in the HOMA-IR and Matsuda index among all three groups were not significant (*P* > 0.05).

**Fig. 1:**
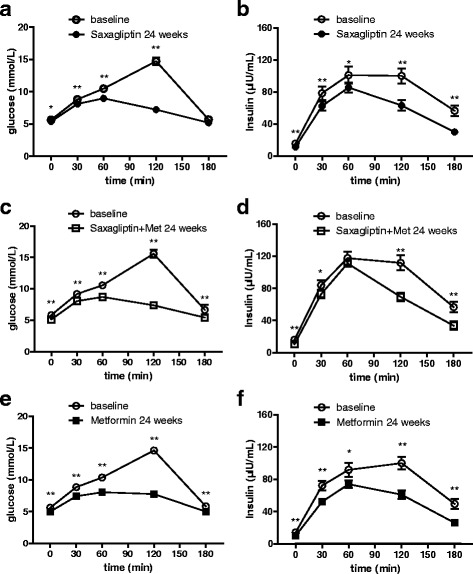
OGTT-based glucose and insulin concentrations before and after treatment in PCOS patients with T2DM. **a, b** Glucose and insulin concentrations based on the OGTT in the saxagliptin group. **c, d** Glucose and insulin concentrations based on the OGTT in the saxagliptin + metformin group. **e, f** Glucose and insulin concentrations based on the OGTT in the metformin group. Data are presented as mean ± SEM. The AUC glucose and AUC insulin are shown in each figure. The *P*-values are based on the Wilcoxon signed-rank test for differences between groups. *P < 0.05; **P < 0.01. OGTT: oral glucose tolerance test; PCOS: polycystic ovary syndrome; T2DM: type 2 diabetes mellitus

In the OGTT, glucose levels were significantly reduced in all three groups at 0, 30, 60, and 120 min following the 24-week treatment (*P* < 0.05 for all). Glucose levels at 180 min in the combination and metformin groups showed a significant decline (combination vs. metformin: − 1.24 vs. -0.83 mmol/L, *P* = 0.001 and 0.009, respectively); whereas in the saxagliptin group, this decline showed no significance (*P* = 0.102). Moreover, after 24 weeks of treatment, all three groups showed a significant decline in insulin levels at 0, 30, 120, and 180 min (*P* < 0.01 for all). Patients in the saxagliptin and metformin groups had significantly reduced insulin levels at 60 min (saxagliptin and metformin: − 15.49 and − 17.88 μIU/mL, *P* = 0.042 and 0.027, respectively). The differences in the AUC glucose and AUC insulin in all three groups were significant, compared to those evaluated 24 weeks earlier (*P* < 0.001 for all). Interestingly, the AUC insulin in the saxagliptin group showed significant improvement, but the FINS showed a greater decline in the metformin group (Fig. 1).

### Changes in parameters of the lipid profile and inflammation after saxagliptin, metformin, or combination treatment in patients with new-onset T2DM

As shown in Table 3, TG, LDL-C, and hsCRP levels in the saxagliptin, metformin, and combination groups were all significantly reduced after the 24-week treatment, compared to baseline levels (saxagliptin group: *P* < 0.001, *P* = 0.046, and *P* < 0.001, respectively; combination group: *P* < 0.001 for all; metformin group: *P* < 0.001 for all). However, among the three groups, the metformin and combination groups showed significant reductions in TC (*P* < 0.001 and *P* = 0.001, respectively), whereas the saxagliptin group showed similar TC levels before and after treatment (*P* = 0.223). Regarding the HDL-C levels, a significant increase was observed in patients of the metformin group after the 24-week treatment (*P* = 0.031). Significant differences in TC and LDL-C levels were observed among the three groups (saxagliptin vs. combination vs. metformin groups: TC: − 0.09 vs. -0.27 vs. -0.38 mmol/L; LDL-C: -0.19 vs. -0.42 vs. -0.43 mmol/L; *P* = 0.005 and 0.027, respectively). In further comparisons between the monotherapy treatments, the effects of metformin were superior to those of saxagliptin in modulating TC and LDL-C levels (*P* = 0.002 and 0.014, respectively).

**Table 3:** Lipid profile and inflammation before and after treatment in PCOS patients with new-onset T2DM

Parameters	Saxagliptin			Saxagliptin + Metformin			Metformin		
	Baseline	Treatment	Δ	Baseline	Treatment	Δ	Baseline	Treatment	Δ
TG, mmol/L	1.44 (1.16 to 1.72)	1.01 (0.92 to 1.09)**	− 0.43 (− 0.67 to − 0.2)	1.34 (1.19 to 1.5)	0.95 (0.86 to 1.04)**	− 0.39 (− 0.53 to − 0.25)	1.32 (1.05 to 1.59)	0.9 (0.79 to 1)**	−0.43 (− 0.62 to − 0.23)
TC, mmol/L	4.51 (4.2 to 4.81)	4.41 (4.19 to 4.64)	− 0.09 (− 0.21 to 0.02)**	4.8 (4.44 to 5.15)	4.52 (4.29 to − 4.75)**	−0.27 (− 0.5 to − 0.05)**	4.94 (4.57- to 5.31)	4.56 (4.28 to 4.84)**	−0.38 (− 0.54 to − 0.21)**
HDL-C, mmol/L	1.26 (1.16 to 1.37)	1.3 (1.24 to 1.36)	0.04 (− 0.03 to 0.1)	1.24 (1.14 to 1.35)	1.32 (1.23 to 1.4)	0.07 (−0.03 to 0.18)	1.35 (1.26 to 1.43)	1.41 (1.35 to 1.46)**	0.06 (0 to 0.12)
LDL-C, mmol/L	3.06 ± 0.7	2.87 ± 0.48*	−0.19 ± 0.36*	3.4 ± 0.73	2.98 ± 0.41**	− 0.42 ± 0.46*	3.32 ± 0.69	2.89 ± 0.45**	− 0.43 ± 0.35*
hsCRP, mg/L	3.97 (3.1 to 4.84)	2.23 (1.84 to 2.63)**	−1.74 (− 2.44 to − 1.03)	3.94 (3.09 to 4.8)	2.51 (2.22 to 2.81)**	−1.43 (− 2.08 to − 0.78)	4.02 (3.23 to 4.8)	3.03 (2.57 to 3.49)**	−0.99 (− 1.44 to − 0.54)

Data are presented as mean ± SD or mean (95% CI). *P-*values based on the paired *t*-test for variables of normal distribution and the Wilcoxon signed-rank test for variables of skewed distribution, for differences between baseline and after the 24-week treatment; one-way ANOVA for variables of normal distribution and the Kruskal–Wallis test for variables of skewed distribution for differences among three groups. Δ denotes the changes after treatment compared with baseline. * *P* < 0.05 and ** *P* < 0.01 for changes before and after treatment in treatment columns, and for changes among three groups in Δ columns

*TG* triglyceride, *TC* total cholesterol, *HDL-C* high-density lipoprotein cholesterol, *LDL-C* low-density lipoprotein cholesterol, *hsCRP* high-sensitivity C-reactive protein

### Changes in anthropometric measurements after saxagliptin, metformin, or combination treatment in patients with new-onset T2DM

Table 4 shows the significant reductions observed in body weight, BMI, WC, WHR, and FAT% after saxagliptin, metformin, and combination treatments, in comparison to the respective values before treatment (*P* < 0.01 for all). Significant differences were observed in the reduction of weight, BMI, and FAT% among all three groups and between the two monotherapy groups (saxagliptin group vs. combination group vs. metformin group: weight: *P* < 0.001, BMI: *P* < 0.001, FAT%: *P* = 0.026; saxagliptin group vs. metformin group: weight: *P* < 0.001, BMI: P < 0.001, FAT%: *P* = 0.043). However, no significant differences were noted in reductions of the WC and WHR among the three groups at 24 weeks (*P* = 0.137 and 0.161, respectively).

**Table 4:** Anthropometric measurements before and after treatment in PCOS patients

Parameters	Saxagliptin			Saxagliptin + Metformin			Metformin		
	Baseline	Treatment	Δ	Baseline	Treatment	Δ	Baseline	Treatment	Δ
Weight, kg	70.4 (63.7 to 77.1)	69.4 (62.8 to 76.1)**	−1.0 (− 1.3 to − 0.6)**	69.3 (64.6 to 74.1)	67.0 (62.4 to 71.6)**	− 2.4 (− 2.8 to 2.0)**	67.9 (63.6 to 72.2)	65.1 (61.0 to 69.3)**	−2.8 (− 3.4 to − 2.2)**
BMI, kg/m^2^	27.2 (24.94 to 29.46)	26.68 (24.51 to 28.85)**	−0.52 (− 0.82 to − 0.22)**	26.38 (24.66 to 28.1)	25.46 (23.79 to 27.13)**	−0.92 (− 1.08 to − 0.75)**	26.4 (24.63 to 28.18)	25.32 (23.63 to 27.02)**	−1.09 (− 1.32 to − 0.85)**
WC, cm	86.8 (81.2 to 92.4)	84.3 (79.1 to 89.5)**	− 2.5 (− 3.1 to − 1.9)	84.7 (80.0 to 89.4)	81.5 (77.1 to 85.8)**	− 3.2 (− 3.9 to − 2.6)	82.8 (78.9 to 86.6)	79.9 (76.4 to 83.3) **	− 2.9 (− 3.5 to − 2.4)
WHR	0.88 ± 0.08	0.86 ± 0.08**	− 0.02 ± 0.02	0.86 ± 0.06	0.83 ± 0.05**	− 0.03 ± 0.02	0.85 ± 0.06	0.83 ± 0.05**	−0.02 ± 0.01
FAT%	36.13 (32.74 to 39.53)	33.89 (30.89 to 36.88)**	−2.25 (− 2.98 to − 1.52)*	35.12 (32.19 to 38.05)	31.59 (29.01 to 34.16)**	−3.53 (− 4.36 to − 2.71)*	33.6 (31.21 to 35.98)	30.5 (28.34 to 32.67) **	−3.2 (− 3.81 to − 2.59)*

Data are presented as mean ± SD or mean (95% CI). *P*-values based on the paired *t*-test for variables of normal distribution and the Wilcoxon signed-rank test for variables of skewed distribution, for differences between baseline and after the 24-week treatment; one-way ANOVA for variables of normal distribution and the Kruskal–Wallis test for variables of skewed distribution for differences among three groups. Δ denotes the changes after treatment compared with baseline. * *P* < 0.05 and ** *P* < 0.01 for changes before and after treatment in treatment columns, and for changes among three groups in Δ columns

*BMI* body mass index, *WC* waist circumference, *WHR* waist hip ratio, *FAT%* body fat percentage

### Changes in sex hormone levels after saxagliptin, metformin, or combination treatment in patients with new-onset T2DM

Table 5 shows the significant reductions observed in T levels after the saxagliptin, metformin, and combination treatments (*P* = 0.03, 0.02, and 0.013, respectively); whereas FSH levels showed a significant decline after metformin and combination treatments (*P* = 0.009 and *P* < 0.001, respectively). Following administration of the metformin treatment alone, the LH levels were significantly reduced (*P* = 0.04), and the FAI levels showed a decline only after the saxagliptin treatment (*P* = 0.026). No significant differences were observed in the reduction of sex hormone levels between the monotherapy treatments (*P* > 0.05 for all). The saxagliptin treatment yielded greater improvements in T and FAI levels, compared to the combination treatment (T: − 0.52 vs. -0.34 nmol/L, *P* = 0.049; FAI: -6.94 vs. -2.35, *P* = 0.015). Moreover, the metformin treatment yielded a more significant increase in SHBG levels than the combination treatment (4.35 vs. 1.57 nmol/L, *P* = 0.016).

**Table 5:** Sex hormone levels before and after treatment in PCOS patients

Parameters	Saxagliptin			Saxagliptin + Metformin			Metformin		
	Baseline	Treatment	Δ	Baseline	Treatment	Δ	Baseline	Treatment	Δ
LH, IU/L	10.01 (6.21 to 13.81)	7.8 (7 to 8.61)	−2.21 (− 5.54 to 1.12)	9.62 (7.24 to 12.01)	7.68 (6.86 to 8.5)	−1.94 (− 4.27 to 0.39)	11.74 (8.59 to 14.89)	7.55 (6.65 to 8.45)*	−4.19 (− 7.32 to − 1.05)
FSH, IU/L	6.27 (5.24 to 7.3)	5.66 (5.16 to 6.16)	− 0.61 (− 1.62 to 0.4)	7.66 (6.94 to 8.38)	5.97 (5.57 to 6.37)**	− 1.69 (− 2.55 to − 0.83)	8.18 (6.83 to 9.52)	5.92 (5.31 to 6.53)**	− 2.26 (− 3.86 to − 0.66)
T, nmol/L	2.64 (2.33 to 2.96)	2.13 (1.94 to 2.32)**	− 0.52 (− 0.69 to − 0.34)*	2.61 (2.35 to 2.86)	2.27 (2.09 to 2.45)*	−0.34 (− 0.52 to − 0.16)*	2.6 (2.33 to 2.86)	2.11 (1.97 to 2.26)**	−0.48 (− 0.65 to − 0.31)
SHBG, nmol/L	24.72 (17.14 to 32.29)	29.62 (23.77 to 35.48)	4.91 (1.56 to 8.25)	25.51 (19.98 to 31.04)	27.09 (22.76 to 31.41)	1.57 (−0.8 to 3.95)*	22.64 (15.73 to 29.54)	26.98 (21.55 to 32.42)	4.35 (1.72 to 6.97)*
FAI	15.82 (11.06 to 20.58)	8.88 (6.4 to 11.35)*	−6.94 (− 9.83 to − 4.06)*	11.72 (8.36 to 15.08)	9.37 (7.57 to 11.16)	−2.35 (− 5.72 to 1.01)*	14.84 (9.06 to 20.63)	9.13 (7.38 to 10.88)	−5.72 (− 10.79 to −0.64)

Data are presented as mean ± SD and mean (95% CI). *P*-values based on the paired *t*-test for variables of normal distribution and the Wilcoxon signed-rank test for variables of skewed distribution, for differences between baseline and the 24-week treatment; one-way ANOVA for variables of normal distribution and the Kruskal–Wallis test for variables of skewed distribution, for differences among three groups. Δ denotes the changes after treatment compared with baseline. * *P* < 0.05 and ** *P* < 0.01 for changes before and after treatment in treatment columns, and for changes among three groups in Δ columns

*LH* luteinizing hormone, *FSH* follicle-stimulating hormone, *T* total testosterone, *SHBG* sex hormone binding globulin, *FAI* Free androgen index

## Discussion

The main findings of this study included the effects of saxagliptin to reduce glucose levels and improve β-cell function and their similarity to the effects of metformin in newly diagnosed patients with T2DM and PCOS. The HbA1c levels showed decline in all three groups after the 24-week treatment. The reduction in HbA1c was significant in the combination group, compared to the monotherapy groups, whereas differences between the monotherapies were not significant. Furthermore, saxagliptin, metformin, and the combination treatment significantly reduced HOMA-IR and increased DI levels, whereas no significant changes were observed in the HOMA-IS of the metformin and combination groups, nor in the insulinogenic index of all three groups. In addition, saxagliptin and metformin treatments significantly reduced the BMI and hsCRP levels.

Impaired secretion and activity of the incretin hormone has been reported in women with PCOS, although the data are not consistent [14–16]. Vrbikova et al. [14] evaluated the relationship between incretin secretion and β-cell function in PCOS. They demonstrated that increased levels of total gastric inhibitory polypeptide (GIP) and lower concentrations of late phase active glucagon-like peptide-1 (GLP-1) were common characteristics observed during the OGTT in women with PCOS, who had higher levels of C-peptide secretion in comparison to healthy controls. Their study suggests that these peptides might be early markers of a pre-diabetic state [14]. Moreover, our previous study [5] showed that impaired β-cell function induced a primary defect in Chinese women with PCOS. It also suggested that impaired β-cell function in PCOS with T2DM might pose a more serious condition than that of those non-PCOS women with T2DM.

Studies in cell cultures and animal models have demonstrated that DPP-4 inhibitors have trophic effects on pancreatic β-cells [17–19] and can improve other metabolic characteristics, such as hyperlipidemia and low-grade inflammation. However, whether DPP-4 inhibitors play a unique role in women with T2DM and PCOS remains unclear. In the present study, we found that the effect of saxagliptin to reduce glucose levels was similar to that of metformin in newly diagnosed patients with T2DM and PCOS. The mean Matsuda index values, whole-body insulin sensitivity evaluation derived from OGTTs, weight, lipid profile, and inflammation showed significant improvement after the 24-week saxagliptin treatment. Notably, we found that the reduction in HbA1c levels was significantly greater in the combination group, in comparison to the other groups of women with T2DM and PCOS. These enhanced effects of the combination therapy to reduce HbA1c levels suggest that β-cell dysfunction has a considerable impact on hyperglycemia in women with T2DM and PCOS in China. In a recent study, the effects of saxagliptin, metformin, and their combination were explored in pre-diabetic women with PCOS [20]. The combination treatment was found to be more effective at improving the insulin secretion-sensitivity index (IS-SI, which was derived by applying the concept of the DI to measurements obtained during the 2-h OGTT) in pre-diabetic women with PCOS [20]. In our study and study by Elkind-Hirsch et al., lipid parameters, such as TG, as well as blood glucose were found to be reduced after saxagliptin and combination treatment. Thus, DPP-4 inhibitors evidently have a beneficial effect on metabolic disorders in both pre-diabetic and diabetic women with PCOS, especially if it is administered in combination with metformin.

When considered together, the above data infer that saxagliptin might be another favorable option to improve insulin sensitivity and sustain glycemic control in women with PCOS and T2DM. The mechanism by which these effects occur might be related to the activation of incretin and the increase in pancreatic β-cell insulin production.

In the present study, changes in the lipid profile (reduced TG and LDL-C levels) and reduced inflammation were both observed after all three treatments. Moreover, reductions were also observed in anthropometric measurements, such as weight, BMI, WC, WHR, and FAT%.

Metformin might be the most effective in long-term maintenance of PCOS, and it might exhibit favorable effects in preventing the progression to diabetes. However, the most common adverse reactions of metformin, the gastrointestinal symptoms (such as diarrhea, nausea, vomiting, abdominal bloating, flatulence, and anorexia), could limit its use in metformin-intolerant patients. Our previous findings suggest that the defect in ß-cell compensation for ambient IR, particularly in the stimulated state, already exists in women with PCOS. With respect to fasting glucose control, metformin treatment is prior to saxagliptin treatment. However, metformin monotherapy might be inadequate for 2-h control of glucose levels.

The conditions of T2DM and PCOS have special characteristics among different ethnic groups. In East Asians, T2DM is characterized by β-cell dysfunction, as opposed to IR due to increased adiposity. Thus, a preventative and therapeutic approach that precisely targets β-cell dysfunction is required [21]. As a result, the fact that saxagliptin enhances the glucose-dependent release of insulin by β-cells makes it an optimal choice for the treatment of T2DM in East Asians. Asian women with PCOS are no more likely to be obese than those without PCOS; however, when present, obesity still has metabolic effects [22]. Moreover, as women of some non-Caucasian ethnicities appear to have higher metabolic risks at a given adiposity, lower BMI and WC targets might be prudent in high-risk ethnic groups [22]. Thus, the effect of metformin on weight loss, as well as its ability to improve the uptake and utilization of glucose in peripheral tissue makes it an optimal choice for the treatment of PCOS in non-Caucasian ethnicities. Therefore, the combination of metformin and saxagliptin might have complementary effects on the treatment of patients with new-onset T2DM and PCOS.

Several limitations of the present study should be considered. Firstly, OGTT is less reliable than intravenous tests, possibly due to the increasing variability of DIx values (DI calculated by various methods). Nevertheless, the OGTT yields more favorable physiological expressions than those of intravenous tests, particularly because ubiquitous glucose sensors could actively participate in insulin activation and secretion [23]. Secondly, the samples of this study were relatively small and its duration was relatively short. Larger sample sizes and studies conducted over longer periods are required for future study. Finally, causality cannot be established with the cross-sectional design of the present study.

## Conclusions

Both saxagliptin and metformin monotherapy treatments were effective in reducing blood glucose and HbA1c levels in women with PCOS and new-onset T2DM. It might be beneficial, during the earlier stages, to add a DPP-4 inhibitor to the treatment protocol for women with PCOS and T2DM.

## Additional file

Additional file 1: Flow diagram of participants. Chart showing the number of participants who were randomly assigned, those who received the intended treatment, and those who were analyzed for the primary outcome. (EPS 1314 kb)

## Abbreviations

AUC glucose: Area under the curve during OGTT performance for glucose
AUC insulin: Area under the curve during OGTT performance for insulin
BMI: Body mass index
CV: Coefficients of variation
DI: Deposition index
FAI: Free androgen indexes
FAT: Body fat
FBG: Fasting blood glucose
FINS: Fasting insulin
FSH: Follicle-stimulating hormone
GIP: Gastric inhibitory polypeptide
GLP-1: Glucagon-like peptide-1
HC: Hip circumference
HDL-C: High-density lipoprotein cholesterol
HOMA-IR: Homeostasis model assessment insulin resistance index
HOMA-IS: Homeostasis model assessment β cell function
IGT: Impaired glucose tolerance
IR: Insulin resistance
LDL-C: Low-density lipoprotein cholesterol
LH: Luteinizing hormone
OGTT: Oral glucose tolerance test
PCOS: Polycystic ovary syndrome
SHBG: Sex hormone binding globulin
T: Testosterone
T2DM: Type 2 diabetes mellitus
TC: Total cholesterol
TG: Triglyceride
WC: Waist circumference
WHR: Waist hip ratio
